# Exploring the Relationship Balance Assessment

**DOI:** 10.1007/s10591-017-9421-2

**Published:** 2017-07-20

**Authors:** Thomas B. Luttrell, Brian Distelberg, Colwick Wilson, Carmen Knudson-Martin, Mary Moline

**Affiliations:** 1 Loma Linda University, Loma Linda, CA USA; 2 Kettering Medical Center, Kettering, OH USA; 3 Lewis & Clark College, Portland, OR USA

**Keywords:** Couples, Equality, Gendered power, Gender roles, Dyadic assessment, Exploratory factor analysis

## Abstract

Assessments of power in couples’ relationships often only survey one partner, but they do not take into consideration both partners’ perceptions. Thus, many assumptions about power and equality in relationships have not been quantitatively tested due to a lack of dyadic measures of power. Therefore, the purpose of the Gender and Relationships Study was to develop and test a new scale of equality and relative power for couples, the Relationship Balance Assessment (RBA). The RBA may be useful for research and for clinical work with couples to help raise awareness of the balance of power in their relationship. A review of the literature has shown a shift away from focusing on monetary resources and decision-making dominance towards examining relationship processes and the connection between gender and power. This study prescreened a pool of process-oriented questions based on the qualitative literature. Then exploratory factor analysis of data from 268 individuals and 91 couples identified 12 consistent latent factors underlying relationship equality. These 12 subscales are summed up with the TREASURES acronym: Time Discretion, Relational Power, Emotional Power (Emotional Expression and Avoidance subscales), Accommodation, Spending and Saving subscales, Union or Sexual Dominance, Rational Power, Economic Role Power (Status and Childcare subscales), and Social Choices.

## Introduction

Marriage and family therapists working with couples have increasingly found that equality or equal power is important for fostering trust and happiness in intimate relationships (Gottman 2011). While there has been plenty of qualitative research that describes relationship power and equality, there are few scales that measure equality or balance that are sensitive to gendered power. As the first phase of the larger Gender and Relationships Study, the purpose of this article is to focus on describing the development of an empirical, clinical assessment of relationship equality. The Relationship Balance Assessment (RBA) is designed to be a reliable measure of relationship equality that can be employed by therapists or researchers.

One of the challenges to measuring equality is that most people typically assess their own relationships as equal, which makes it more difficult to measure (Steil 1997). Those with more power are usually unaware of it. Inequality is difficult to detect or observe directly and is often a result of the social context. Therefore, asking for one partner’s perception may or may not reflect the power imbalance of a couple’s relationship. By studying both partners, we were able to identify questions that are sensitive enough to assess the balance of power for both men and women, so that we can see how gender plays a role.

Furthermore, because couples may find it difficult to critically answer direct questions about power, a factor analysis helps establish common factors within relationship power to guide assessment. So far, few have quantitatively differentiated the components of relationship equality. While past models tended to view the balance of relationships as unidimensional, there has been a growing awareness that balance in relationships is multidimensional. Relationship balance has been analyzed in terms of cohesion and adaptability or in terms of domains of conflict areas (Godwin and Scanzoni 1989a; Kurdek 1994; Olson et al. 1979).

To be clear, the purpose of this study is *not* to identify all the possible areas in a relationship that are affected by power imbalances. The possible consequences could be varied and unlimited due to differing cultures. Rather, the purpose is to identify those common areas in which power imbalances are relatively easy for couples and professionals to discern or detect. Using exploratory factor analysis (EFA), this study identified potential factors in relationship equality in order to create an assessment that helps couples see their own relationship balance. EFA was conducted across different levels to see how factors compare between couple and individual responses. The purpose was to develop and test an assessment that is reliable on either the individual or couple level, and to identify factors that can be correlated with outcomes.

## Power as Decision-Making Dominance

Early approaches to power used social exchange theory to conceptualize relationship power as decision-making dominance, relative to another person (Kulik 2011). The concept of power came from the *conflict theory* of Marx and Engels. Power imbalances result when people have control over resources that others need, and those with power have the ability to get what they want (Parrado et al. 2005; Shields 2000; White and Klein 2008).

Past studies of partner dominance focused on who dominated decision-making and division of labor. A common assumption was that money was the basis for power (Blumberg and Coleman 1989). Early research on heterosexual couples looked at the amount of time women worked or the amount of money she earned. As women earn more money, they may have more decision-making power, perhaps because they are not as dependent on their partner (Blumberg and Coleman 1989; Parrado et al. 2005). Perceptions of equity and fairness also appear related to resources, time, and power in the division of labor (Sanchez and Kane 1996).

Social exchange theory has been criticized for overlooking the social context, in particular gender and class, that shapes the value of resources partners contribute (Nakonezny and Denton 2008; Parrado et al. 2005). Thus, a *feminist* framework shifts the focus from economic resources to gender as a source of power and inequity (White and Klein 2008). Thus it is often called *gendered power*.

Money or economic factors alone are not enough to account for imbalances in relationships, as gender provides a more accurate and nuanced understanding (Britt and Roy 2013). Multiple studies found that as women earned more money, they still did more housework and their influence in decisions tended to not increase at the same rate as men. Partners frequently maintain male power, in effect compensating for his lesser income (Kamo 1988; Kulik 2011; Steil 1997; Stuchell 2013; Tichenor 2005). A study comparing stay-at-home fathers and stay-at-home mothers found that they experienced their roles very differently due to social expectations for men and women (Zimmerman 2000). Thus, stay-at-home mothers did more parenting or childcare and were more exhausted than stay-at-home fathers, who often saw their situation as temporary (Zimmerman 2000).

Decision-making dominance may be deceiving as a measure of equality or power, because some areas may be delegated based on what the other partner does not want responsibility for or because some tasks are too tiring or less important (Blumberg and Coleman 1989; Steil 1997). While they are often related, the one who usually has more say in particular decisions may not always be the one with more power (Hahm et al. 2012; Pulerwitz et al. 2000). In fact, a *perception* of power may be based only on how the couple assigns responsibilities (Kamo 1988). For example, a woman may be cast as having power over how their children are raised, but it also could be that the man delegates these responsibilities to her as he maintains power in other areas. In addition, one partner’s power does not necessarily mean the loss of power for the other partner (Dunbar and Burgoon 2005).

## Equality as a Mutual, Dyadic Process Rather than Individual Dominance

### Systemic Conceptualization

For this study, power and equality are conceptualized as systemic, using systems theory, and dyadic, meaning it requires at least two parties (Dunbar 2004). This means that both partners influence each other in some way and co-organize the structure of a relationship (Dickerson 2010; Sutherland and Jeffrey 2016; White and Klein 2008). The few quantitative studies of power, such as those studying dominance in sexual decision-making (e.g., Pulerwitz et al. 2000), tend to survey only women for their perception of power, which overlooks how their power may be relative to their partners’ power (Blanc 2001). In addition, looking at only one person or their average view may hide divergences or differences between partners. Individual perceptions cannot be aggregated (de Palma et al. 2011). An assessment of power *must* involve comparing both partners’ perceptions, because assessing only one partner’s perception cannot adequately capture all of the dimensions of power.

### Invisible Dimensions of Power

In a relationship, *manifest power* may be visibly seen as a person’s ability to influence the other person or the amount of dominance or control they have in decisions (Dunbar 2004; Komter 1989; Steil 1997). Yet, power is not typically visible or evident until changes are sought or conflicts arise. *Invisible power* is seen in unequal levels of esteem or subtle differences in perception, such as when husbands underestimate their wife’s share in household tasks or child care, while overestimating their own contributions (Komter 1989). Inequality is often maintained by invisible processes, such as social norms about what it means to be a man or woman, what is masculine or feminine, and how men and women interact (Komter 1989).

### Equality as Mutual Process

A mutual relationship is one in which both partners are equally valued. They are mutually open to influence, are willing to be vulnerable, care for and respect each other, and share a sense of responsibility for maintaining the relationship (Steil 1997). Grounded theory analysis by Knudson-Martin (2013) has identified patterns that promote equality or mutual support: mutual couples had a shared sense of relationship responsibility, mutual vulnerability, mutual attunement, and mutual influence. *Attunement*, a process of being relationally present and aware of the needs of the other, was key and connected to equal intentionality, continual communication, partnership, mutual understanding and joint decision-making (Jonathan and Knudson-Martin 2012). Mutuality is important, even in hierarchical societies (Moghadam et al. 2009; Quek et al. 2009).

### Establishing Objective Criteria

This study looks at power and equality as a systemic, relational process, where partners mutually influence each other. Invisible power can be made visible by looking at how partners are seen and heard, and in how partners are able to disagree and still value each other. Equality and mutuality require that people look beyond each other’s social roles and see the inherent value and equal worth in each other and respond in ways that demonstrate it. Because mutuality is more subjectively defined and most couples will say they equally value each other, it is important to identify more objective, behavioral criteria that can help raise awareness about the gaps between ideals and reality (Knudson-Martin and Mahoney 1998; Steil 1997). The literature suggests a hypothesis that there are multiple common factors of power or relationship balance. The following section contains a table (Table 1) of all the factors used in the development of the Relationship Balance Assessment (RBA), along with references.

**Table 1:** Previous research on items in the original RBA question pool

Aspects of balance	Literature
Ability to influence	Gottman (2011); Knudson-Martin (2013); Komter (1989); Steil (1997)
Accommodation	Cheung 2005; Jonathan and Knudson-Martin (2012)
Appreciation	Britt and Roy (2013); Lee and Waite (2010); Wilcox and Nock (2006)
Assertiveness	Gottman (2011); Knudson-Martin and Mahoney (2005); Knudson-Martin (2013)
Attunement	Jonathan and Knudson-Martin (2012); Knudson-Martin (2013)
Challenging entitlement	Knudson-Martin and Mahoney (2005)
Compromise	Cheung (2005); Quek and Knudson-Martin (2008)
Equity or fairness	Bahmani et al. (2013); DeMaris (2007)
Intentionality	Jonathan and Knudson-Martin (2012)
Investment	Britt and Roy (2013); Sprecher et al. (2006); Steil (1997); Waller (1938)
Involvement in decisions	Blumberg and Coleman (1989); Harvey et al. (2002); Jonathan and Knudson-Martin (2012); Komter (1989)
Listening	Jonathan and Knudson-Martin (2012); Knudson-Martin (2013); Williams and Knudson-Martin (2013)
Monetary resources	Bahmani et al. (2013); Kan (2008); Kulik (2011); Oreffice (2011); Schwartz et al. (1995); Steil (1997); Stuchell (2013); Tichenor (2005)
Negotiation	Jonathan and Knudson-Martin (2012); Kulik (2011); Knudson-Martin and Mahoney (2005); Quek et al. (2009); Steil (1997)
Openness to Influence	Knudson-Martin (2013)
Partnership	Jonathan and Knudson-Martin (2012); Nakonezny and Denton (2008)
Quality time	Lee and Waite (2010); Wilcox and Nock (2006)
Relational responsibility	Knudson-Martin (2013)
Respect	Bahmani et al. (2013); Knudson-Martin (2013); Moghadam et al. (2009); Quek et al. (2009); Steil (1997)
Sexual desire	Schnarch (1991)
Sexual roles	Schwartz et al. (1995)
Social support and status	De Jong (2000); Harvey et al. (2002); Schwartz et al. (1995); Smits et al. (2003)
Understanding	Jonathan and Knudson-Martin (2012)
Validation	Knudson-Martin (2013); Williams and Knudson-Martin (2013)
Value	Nakonezny and Denton (2008); Sprecher et al. (2006); Steil (1997); Waller (1938)
Vulnerability	Holm et al. (2001); Knudson-Martin (2013); Knudson-Martin and Mahoney (2005); Mirgain and Cordova (2007); Schnarch (1991)

## Methods

### Research Design

The purpose of this cross-sectional study was to create a measure that can be used by clinicians to assess equality in couple relationships and account for the gendered nature of power. The first goal is to find factors where couples could readily perceive the balance of power for themselves. Exploratory factor analysis (EFA) was conducted on the Relationship Balance Assessment at the individual level and couple level. The protocol and survey was reviewed and approved by the Loma Linda University Institutional Review Board (IRB #5140217).

#### Recruitment

Groups of participants were recruited through three methods. In the first method, couples over the age of 18 were recruited through online advertisements on counseling-related groups on Facebook, the social media website. In the second method, adult couples were recruited through workshops at churches in the area that the study researchers had access to. Researchers also asked professional therapists to invite couples within their practices.

#### Data Collection

Surveys were conducted via a paper version and an online version. The surveys used five-digit identification numbers in sets of two to match partners with each other. The paper survey was returned to the researchers in a sealed business reply envelope, so that there was no way to concretely identify participants.

#### Participants

Participants in this study were adult (18+) couples who believed they were in a committed relationship. To be included in this dyadic study, both partners needed to participate; however, if one partner did not participate, their responses were still used for comparison between dyadic and individual assessments. Four gay and lesbian participants were excluded from couple-level analysis since the focus was on heterosexual couples for comparison purposes. However, their information was retained on the individual level because there were many participants who did not have matched partners. While 87% of participants came from the United States (N = 232), some came from Canada (N = 19), Australia (N = 4), and other countries (N = 8). Because of the recruitment strategy, 62% of participants (N = 165) were from California where the research originated.

Overall, there were 268 individual participants. A small group of 22 people (8%) consisted of a clinical population of participants who were referred by a participating professional counselor or therapist when it was felt that an assessment of relationship balance could be beneficial to a client. A second population consisted of mostly Seventh-day Adventist participants. About 21% (*n* = 58) reported that they were referred through a church-based workshop or seminar. A third population consisted of couples across the country recruited through friends, family and Internet advertising.

### Measures

The survey for this study asked for basic information about participants, such as their sex, age, racial or ethnic category, relationship status, and duration of relationship. The study also included other scales that will be explored in later phases.

*The Relationship Balance Assessment (RBA)* is a dyadic relationship assessment created for this study based on the literature about different domains of gendered power in relationships. Because this study sought to establish behavioral criteria that couples could identify for themselves, multiple factors were considered. The study looked at different theory-based domains: resources, ideology, division of family labor, mutuality processes, equity or relative fairness, emotional intimacy, and sexual intimacy. The balance of mutual emotional processes includes Knudson-Martin and Mahoney’s (2005) concepts of mutual support and shared vulnerability. Questions related to gender ideology (Kulik 2011; Steil 1997; Stuchell 2013; Tichenor 2005) were separated into a different scale. Division of family labor (Kan 2008) was also looked at as a separate variable in relationship to the RBA. Table 1 summarizes factors that were drawn from the literature for the development of the assessment. At least five questions were written per hypothesized domain.

The RBA contained a list of questions that asked participants to evaluate who benefits more, following a polarized approach that requires them to choose between either partner. The answers are Likert-scales that have both partners on one continuum, with “Equal” in the middle. However, it also measures the extent to which partners report a balance between them when they select Neutral/Equal.

### Pre-analysis

The Relationship Balance Assessment went through a process of pilot-testing, data collection, reduction, and analysis. After collecting data, data were screened and then assumptions were tested before conducting exploratory factor analysis (EFA). Specifically, EFA models were generated for men, women, and couple averages and differences. The scale was reduced even further based on factor loadings for items across the models, and then factors were identified and labeled.

#### Pilot-Testing and Reduction

The first draft of the RBA originally contained 137 questions measuring relationship balance, in addition to the other sections of the survey. Due to the length, the first draft was pilot-tested on a small group of 29 individuals to determine which questions to remove based on their usefulness. Based on these early results, items were screened for outliers and tested for the assumptions that all variables are normally distributed, by looking at their skewness and kurtosis (Mertler and Vannatta 2010). Since the goal of the study is to identify which questions were most predictive of the balance of power, those questions that were more commonly endorsed as “equal” (had a high kurtosis) were removed since they would not be sensitive enough. Some of the original items that the vast majority of early respondents said they were “equal” on included: who had resources; who took responsibility or was committed to the relationship; who had a sense of partnership; who was vulnerable and shared their struggles; who was willing to negotiate; who asserted their emotional or spiritual needs; who compromised more in religion or with parents/in-laws or with finances; who had the final say in important decisions about family life, living together or having a baby; who put the other first or accommodated the other more; who influenced the other’s thoughts or feelings or the other’s family life or career decisions; who was more attuned or responsive to the other’s needs; who got their needs met more; who was more open to learning and being influenced; who was more entitled; and who was more valued.

In addition, some questions were removed if they were extremely skewed toward one person or another (skew >1.5 or <−1.5 or had means <2 or >8), which means that partners tended to answer “myself” or “my partner.” While these questions may be perfectly valid and legitimate questions to ask, the concern is that they also may be more prone to being answered according to endorsement of perceived gender stereotypes, more than their actual balance of power. Their removal does not indicate that they are not important, but rather preference for items that have a more normal “bell curve” distribution with more variability in order to be sensitive enough for the uniqueness of each couple. The analytic methods used also require normal distributions; however, few items were removed based on skewness alone.

This helped reduce the number of the RBA’s questions for the main data collection by over half. About 60 remaining items were chosen based on which ones had more variability and a more normal distribution. After the RBA was reduced, more couples were recruited to take the survey online and via a paper survey, as described above. The survey included the reduced RBA, along with other measures of gender and brief measures for relationship, sexual and life satisfaction that are outside the scope of this article.

#### Missing Data

A process for handling missing data was implemented. Participants with a high rate of more than 20% skipped or missing questions were considered for exclusion, and then items with a poor response rate (skipped by 5% of remaining participants) were dropped. After that, any remaining missing data were filled in by imputing the estimated means for each item by gender (Cohen et al. 2003).

#### Assumption Testing

In conducting exploratory factor analysis of the survey data, there are generally some assumptions that must be met. Items were tested for univariate and multivariate normality (Mertler and Vannatta 2010). Tests to determine whether the data were factorable include looking at the Kaiser–Meyer–Olkin (KMO) measure of sampling adequacy, the determinant of the R matrix, and Bartlett’s test of sphericity (Beavers et al. 2013; Field 2009).

### Exploratory Factor Analyses

Exploratory factor analysis (EFA) was conducted on both individual and couple levels of data to identify the underlying latent factor structure. These models helped to reduce the RBA by highlighting the items that did not load onto a factor or cross-load.

#### Analysis of Men and Women Separately

Exploratory factor analysis was conducted for men and women separately and then both averaged together to see if there is a difference in factor structures between individual and dyadic approaches. Men and women were also separated in order to avoid any issues of redundancy or multicollinearity as many were paired in relationships. Then the factor loadings were compared between men and women.

#### Analysis of Couples Together

Scores for items on the Relationship Balance Assessment were computed for each couple by averaging items for partners. Because the Likert scale questions ask participants to rate whether an item applies to “me,” “my partner,” or either, then one partner’s scores for those questions on the RBA were reverse-scored in order to match ends of the scale. Dyadic-level data were saved in a separate file different from the raw, individual data. An additional analysis was based on the level of *agreement* between partners to see what questions have higher agreement than others. The purpose of this was to look at the different ways items may cluster together based on the differences between male and female partners or on their level of agreement.

#### Communalities

To conduct a factor analysis, items should generally share common variance. *Communalities* are the proportion of shared variability for an item that is explained by the latent factors, or their proportion of common variance. The proportion or communality should be greater than 0.7 (Field 2009), or at least 0.6 with at least four items, for any sample size (Mertler and Vannatta 2010). Thus, a variable that has little in common with other variables would have a low communality. However, it was assumed that this scale may contain factors that do not correlate well with other factors. Because of the composite nature of the Relationship Balance Assessment, communalities were *not solely* relied upon for item reduction. Therefore, items were evaluated based on their factor loadings across multiple models. Nevertheless, if an item had a very low communality (<0.30), then it was considered for removal.

#### Extraction of Factors

The extraction method was common factor analysis, specifically using the Principal Axis Factoring (PAF) method, instead of Principal Component Analysis (PCA), because common factor analysis is more common and appropriate for finalizing scale items (Lee and Lim 2008) and because it was assumed that the RBA contains latent constructs.

To determine the number of factors to retain, the *eigenvalues* for each factor were considered. The eigenvalue is the amount of the total variance explained by each factor, which if added together equals the total number of factors (Mertler and Vannatta 2010). Kaiser’s criteria is that factors should have a value greater than 1.0, though this is more accurate when there are less than 30 original variables and communalities are greater than 0.70, or when the number of participants is greater than 250 and communalities are at least 0.60 (Field 2009; Mertler and Vannatta 2010). Others recommend retaining factors with a cumulative percentage of 70% of the total variability (Field 2009), though some have suggested that as little as 50% can be acceptable (Beavers et al. 2013). Beavers et al. (2013) recommend not basing this on one test, but by varying the number of factors and comparing models to see if they have a clear meaning.

#### Rotation

After the number of factors was determined, the analysis was run with the specified number of factors to extract. The resulting structure matrices were rotated to achieve the best defined factor structure (Field 2009). Because it was assumed that factors correlate with each other, an oblique rotation method was used called Promax rotation.

#### Scale Reduction

Separate exploratory factor analyses (EFA’s) were simultaneously conducted for men and women separately on the individual level of data and for matched couples in order to reduce scale items and then to identify the latent factor structure within the RBA. Scale reduction was accomplished through a series of Principal Axis Factoring factor analysis that helped reduce items based on poor factor loadings and communalities (Mertler and Vannatta 2010). Items with the lowest factor loadings and lowest communalities across all models were removed for having little in common with the rest of the items.

#### Identifying Factors

After well-fitting models were clearly established, the models were compared with each other to see how the factors were similar or different. After factors were consistently identified in each model, descriptive labels were given to the extracted factors based on the scale questions that remained in each factor. Once latent factors were clearly identified for the Relationship Balance Assessment, sub-scale factor scores were calculated for each individual participant, as well as for each couple dyad.

## Results

The primary goal of this study was to create a clinical assessment of relationship balance that was sensitive to the different aspects of power in romantic relationships. Therefore, the study sought to determine the number of reliable factors and to differentiate the latent factors of relationship balance that can be found in the items drawn from the qualitative literature. For the final RBA, 12 factors were extracted that included 35 items. The subscales identified were Relational, Sexual, Emotional, Rational, Spending, Financial Needs, Time, Accommodation, Avoidance, Status, Social and Children.

### Demographic Analysis

Many participating individuals could not get their partner to participate and could not be included in couple analysis. It was harder to recruit men, and as such, at the individual level, men (n = 113) composed 42% of the sample and women made up 58% (n = 155). On the couple level, only 91 heterosexual couples were matched, with 91 men and 91 women.

We found paper surveys far more effective at recruiting *both* partners to participate and able to get a wider range of ages, while the Internet survey captured a younger audience with fewer matched partners. Participants using the paper survey were on average 13 years older than online participants, and they had wider variability (SD = 17.3 compared to SD = 14.0, respectively). The differences in age also affect most of their demographic information.

On the individual level, men in the study tended to be older, in relationships longer, having slightly more education, earning around US$20,000 more, and reporting more (+9%) full-time employment. The individual-level sample, regardless of gender, provided a wide range of ages, from 18 to 84 years (Mean = 44.6, SD = 16.5). The mean age for matched couples was 46.3 (SD = 16.8), and they were together an average of 20.9 years (SD = 16.7), with an average of 1.6 children (SD = 1.6). About 72% of partners reported that this was their first marriage, 10% reported that they had been divorced previously, and 18% of participants skipped this.

Across all groups, the majority of individuals and couples had at least a 4-year college degree. The median reported personal income of all participants was US$30,000–$39,999, while the median total family income was US$70,000–$79,999. (Income options were specified in United States currency so that answers would be standardized.) Matched couples reported statistically similar levels of education and household income as unmatched participants, while males had higher education and income than females, as described above.

### Pre-analysis Results

#### Handling Errors and Missing Data

The data set was screened for errors and duplicate records. Couples who did not meet the inclusion criteria were excluded from the couple-level dataset. Forty individuals were excluded for having skipped more than 20% of the items. The remaining missing responses were substituted using a “series mean” method, performed separately by gender. As missing data were not more than 3% per item (with the exception of child care), it was assumed that the series mean imputation was acceptable over other advanced imputation methods (Cohen et al. 2003). After imputing series means and removing some items, tests for sample size adequacy and factorability were successful.

#### Assumptions of Normal Distribution

When separating the data by gender, many of the items in the survey were deemed non-normal distributions. Because of the type of dichotomous questions, the relationships tended to have a slight linear curve in scatterplots; yet they were linear enough to produce significant results in subsequent analyses. The primary reason is that the RBA balance-type questions tend to receive high endorsements at the middle range or on the extremes, which resulted in a greater kurtosis or skewness. Because of this, the items with the smallest standard deviation and the least normal distribution, in terms of kurtosis and skewness, were considered for removal. However, to avoid ignoring possible gender differences, items were only considered for removal if they were non-normal for *both* genders. Overall, 5 non-normally distributed scale items were selected for removal from the RBA (who listened more, who respected in-laws more, who was labeled crazy, who had more to contribute, and who was getting a better deal), while 4 other items were retained because of their predictive ability and factor loadings. A similar process was followed for couple-level data. After items were averaged together to create scores for each couple, 10 non-normally distributed items that were skewed or had a high kurtosis and small standard deviation were selected for removal, while 3 other items were retained because of their predictive ability and factor loadings.

### Exploratory Factor Analysis and Scale Reduction

After preparing the data, a series of exploratory factor analyses using Principal Axis Factoring and Promax rotation were conducted for items on the Relationship Balance Assessment. EFA models were created for men, women, everyone, and their averages and differences as couples. Items were simultaneously retained or excluded based on their factor loadings across the different models for men, women and couples, using Principal Factor Analysis. After scales were reduced based on their factor loading, the fit of the models were determined according to the assumption tests. As this was a complicated process, the process is summarized briefly due to space limitations.

All of the models were statistically factorable and assumption testing produced positive results, providing confidence in the EFA results despite having only 91 matched couples. A summary of each model’s statistics is presented in Table 2.

**Table 2:** Fit statistics for EFA models

	Women N = 118	Men N = 100		All N = 218	Couple average N = 91		Agreement N = 91	
		Model 1	Model 2		Model 1	Model 2	Raw	Absolute
# of factors	11	9	11	10	10	12	11	8
Items remaining	33	28	28	30	35	33	25	27
Min. eigenvalues	1 or 0.9	1	0.8	0.8	1	0.75	0.8	1
Determinant of R matrix	0.962E−008	1.12E−006	1.12E−006	1.17E−006	6.79E−011	3.44E−010	5.12E−005	9.34E−006
Bartlett’s test of sphericity	p = .000	p = .000	p = .000	p = .000	p = .000	p = .000	p = .000	p = .000
KMO test of sample adequacy	0.602	0.625	0.625	0.736	0.634	0.627	0.524	0.752
% variance explained	60.5%	59.9%	64.9%	60.3%	63.9%	72.3%	62.5%	51%
Residuals	8%	12%	3%	4%	14%	2%	5%	16%

#### Factorability

One of the initial concerns was whether the datasets were factorable. While the determinants of the correlation matrices were initially low, other tests confirmed that the data were factorable. The determinant of the R-matrix for this data tended to have a zero (0) or extremely low value, which suggests that the correlation matrix was close to being singular and perfectly linear (Field 2009). Reducing the number of items increased the value of the determinant. In all factor analysis models, Bartlett’s test of sphericity produced significant test results, with a p = .000, rejecting the null hypothesis, providing evidence that the observed correlation matrices were statistically different from a singular matrix (Beavers et al. 2013). This means that the variables correlate and the correlation matrix is statistically factorable. In addition, the strong consistency of factors across multiple models demonstrates that a clear set of reliable factors do exist that men and women generally agree on and that spans both individual and couple levels.

#### Sample Size Adequacy

There were some initial concerns about whether 218 individuals or 91 couples would be large enough for factor analysis involving 60 variables. On the individual level, there was more than enough of a sample size for a factor analysis. However, using the original data set with missing data, when dividing the data by gender or by couples, the sample size was barely adequate. By imputing estimated, predicted values into missing data based on item means, as described above, the KMO value increased. Therefore, in all factor analysis models, sampling adequacy was usually above >0.60, meaning the sample size was acceptable (see Table 2.)

In the past, the rule of thumb for EFA sample sizes was to have 5–10 participants per variable used (Costello and Osborne 2005). However, strict rules have been abandoned, and in a survey of EFA studies, researchers found that a large majority of studies (63%) are less than the rule of 10:1, and about one-sixth of studies have less than a 2:1 ratio of participants per variable (Costello and Osborne 2005). Newer studies have shown that the quality of data is more important than sample size. Quality data is determined by having high communalities (communalities in social sciences tend to be between 0.4 and 0.7), not having cross-loading variables with loadings greater than 0.32 on more than one factor, and factors of at least three items with greater than 0.50 for factor loadings (Costello and Osborne 2005).

Obviously, larger sample sizes are always encouraged, as the size of the sample likely limited the number of items that could be retained in the final models. However, EFA has been shown to yield reliable results for sample sizes below 50 when certain conditions are met (de Winter et al. 2009). In a review of the literature about EFA, it was found that small sample sizes below 50 were adequate when communalities were high (0.8–0.9) and the number of factors was small. A sample size of 25 was acceptable when loadings were as high as 0.8. Research by de Winter, Dodou and Wieringa (2009) also showed that a larger number of variables can actually improve recovery of factors when loadings are low.

The quality of this study’s data made up for the size of the sample. Factor loadings in this study were strong considering the sample size. Moderate to weak factor loadings ranging between 0.3 and 0.5 are common in social science data (de Winter et al. 2009). Yet, factor loadings for the EFA models presented here generally range from 0.5 to.7, and go as high as 0.9. Lastly, the value of the Kaiser–Meyer–Olkin (KMO) measure of sampling adequacy is reassuring that the sample size was adequate for the study.

### Models of EFA Factors Extracted

When separated by gender, some factors combined together. This was handled by adjusting the minimum eigenvalues lower. For some of the models, the cumulative percentage of factors extracted were increased to >70% when lowering the minimum eigenvalue to less than 1. Usually the method determines the amount of variance that will be explained as component analysis tends to result in higher variance explained than with common factor analysis (Beavers et al. 2013). The oblique Promax rotation also worked well since it allowed for all latent factors to be better represented. After an iterative EFA process that reduced items based on factor loadings, the best fitting models of factors were determined by having a high percentage of variance explained and a low percentage of nonredundant residuals.

For both men and women, the largest factor seemed to have items related to being attuned to others and listening or relating to others. The second factors had items related to sexual dominance or expressing sexual needs.

For comparison purposes, all men and women (N = 218) were combined together in one integrated model to identify the latent structure when all individuals are combined together. Ten factors were extracted that explained about 60% of the variance.

EFA models were created based on the averages and differences of matched couples. The EFA process for couples’ averages was simultaneous and similar to the process at the individual level. Normally-distributed items were reduced based on factor loadings using Principal Axis Factoring and Promax rotation with Kaiser normalization. Item reduction occurred simultaneously with the individual-level data based on items that had the lowest factor loadings across all models, though the results looked slightly different. After items were reduced, there were 35 remaining scores based on couples’ averages (N = 91) (see Table 3).

**Table 3:** Twelve factors extracted for couples (N = 91) after lowering minimum eigenvalue

	1	2	3	4	5	6	7	8	9	10	11	12
55. Time for relationship	0.844											
91. Aware of other’s feelings	0.758											
84. Listened to needs	0.699											
51. Maintains connection	0.695											
69. Negotiates conflict	0.680											
68. Asserts friend needs	0.678											
92. Cared other’s health	0.574											
90. Asked questions	0.515											
83. Influenced sexually		0.955										
65. Express sex needs		0.777										
96. Sexually dominant		0.766										
64. Silent in conflict			0.906									
86. Likely to shut down			0.802									
63. Withheld emotions			0.606									
82. Friend choices				0.780								
78. When seeing family				0.779								
76. Decides friend time				0.667								
75. Final say spending					0.935							
74. Allocated money					0.647							
59. Shared $ concerns						0.854						
67. Asserts $ needs						0.821						
95. Time discretion							0.939					
94. Time for interests							0.796					
89. Used rationality								0.790				
88. Considered rational								0.776				
54. Cares 4 sick child									0.782			
53. Time w/ children									0.728			
81. Alter habits 4 other										0.857		
80. Gave in to other’s wishes										0.763		
61. Expressed feelings											0.827	
62. Needed other more											0.549	
45. Higher education												0.898
44. Higher job status												0.402

72.3% of variance is explained with minimum eigenvalue of 0.75

Factor loadings less than 0.4 are suppressed in all EFA tables

Principal Axis Factoring was also conducted on the *differences* between partners’ responses to see if there were latent factors underlying patterns of agreement. This was done using the raw differences, subtracting the female from the husband, and then using the absolute value of those differences. Based on their raw differences, there were some similarities to other models. Latent factors underlying raw differences were easy to interpret as they were were somewhat similar to factors based on the average scores for couples. Factors that had either a high level of agreement, such as relational or social constructs, or a high level of disagreement, such as time or accommodation, were both present and easily discernible. However, while a few of the factors were similar-looking, questions related to relationship-orientation, emotional expression and accommodation did not load consistently.

An EFA was also conducted using the absolute value of differences between partners. This measurement is not sensitive to the direction of who benefits or not. Using this method, far fewer items could be extracted using Principal Axis Factoring and many had to be dropped due to poor factor loadings. The remaining items formed into an eight-factor model. Unfortunately, efforts to reduce the high number of residuals only resulted in factors that could not be interpreted or recognized. Latent factors underlying the *absolute* value of agreement or difference scores were not at all similar to the latent factors based on couples’ averages. For example, while sex and money loaded separately in other models based on their perceptions of balance, they loaded together in this model based on how they agree.

### Common Factors Across Models

#### Men Versus Women Versus all Individuals

Perhaps because there were more women in the study, the EFA process for women was able to retain slightly more items than it did for men. Comparing the models helped to reconcile some of the minor differences and to ensure a fair representation of men and women at the couple level. The models for men (N = 100) and women (N = 118) were compared side-by-side with each other and with the combined model with everyone (N = 218). Tables were used to see how they were similar or different.

It was expected that men and women may report some similar and different factors in power. For the most part, men and women surprisingly revealed a lot of similarity in how power is conceptualized. For example, relationship orientation, or other-centeredness, explained the largest variance for *both* genders. However, there were small differences in how men and women perceive power. Despite their lower sample size, items related to expressing feelings and vulnerability loaded strongly together for men, but not for women. On the other side, items related to accommodating their partner loaded consistently for women, but not for men. This does not necessarily mean that men express their feelings more or that women are more accommodating, but rather it also may simply represent a greater sensitivity to how these areas are used.

#### Individual Versus Couple Level

The two models based on the individual and couple levels were also compared side-by-side in a table (not included due to space). For the individual level, the model that represented all men and women individually (N = 281) were used. For the couple level, the model based on the couples’ averages was listed (N = 91). For the most part, the couple’s model was quite similar to those on the individual level. In addition, the models based on couples’ averages and on their agreement were compared side-by-side in a table (not included). About half of the items shared a similar factor structure, though some of the variables based on differences did not load well.

### Creating Subscale Scores

After subscales were clearly defined based on the common factors identified, scores were calculated for each factor based on averaging the value for each item in the subscale, with high scores pointing toward women and low scores pointing toward men. Then, the relationship between each subscale was assessed to determine which direction they were scored. After reversing the Relational, Emotional, Accommodation and Child Care subscales, almost all of the subscales significantly correlated with the full scale score totals with the exception of the Social subscale. Regardless of which direction the Social subscale was scored, it did not correlate significantly with any of the full scale scores for the couple level, perhaps because of the skewness was 1.67 and transformations did not help.

### Reliability of Relationship Balance Assessment

After reversing some subscales, the overall reliability for the entire scale was α = 0.85 on the individual level and α = 0.78 on the couple level. The reliability of the full scale increased to α = 0.80 on the couple level when the Time subscale was excluded. As subscales are present, Cronbach recommended that the reliability for each subscale should be assessed independently (Field 2009). Cronbach’s alpha was calculated for each subscale to see if any item needed to be removed. Because of the method used, no items needed to be removed from any subscales as a result of reliability analysis demonstrating that the subscales were highly consistent and reliable. On the individual level, Cronbach’s alpha ranged from α = 0.627 to 0.837. On the couple level, they ranged from α = 0.675 to 0.868 (see Table 4). While some subscales were less reliable than others, such as the Time subscale, all of them had interesting correlations with relationship outcomes.

**Table 4:** Reliability analysis for relationship balance assessment

Item	Individual		Couple	
	*R*	Alpha if deleted	*R*	Alpha if deleted
Relational (Individual-level α = 0.821. Couple-level α = 0.874.)				
51. Who made active efforts to maintain connection?^a^	0.56	0.799	0.67	0.854
55. Who has given more time to the relationship in general?^a^	0.63	0.791	0.69	0.854
68. Who asserted their needs about friends more?	0.40	0.821	0.59	0.862
69. Who was willing to negotiate when disagreeing about family, sex or parenting?	0.57	0.797	0.61	0.861
84. Who listened more to the other’s needs?^a^	0.60	0.793	0.65	0.857
90. Who proactively asked questions to understand the other?^d^	0.46	0.814	0.54	0.869
91. Who was more aware of the other’s feelings?	0.66	0.782	0.75	0.844
92. Who cared more about the other’s health and well-being?^a^	0.51	0.805	0.59	0.862
Sexual (Individual-level α = 0.825. Couple-level α = 0.850.)				
65. Who expressed their sexual needs more?	0.67	0.793	0.71	0.829
83. Who influenced the other sexually?	0.74	0.728	0.83	0.716
96. Who took the dominant role in sex? (or sexually?)	0.68	0.764	0.68	0.834
Emotional (Individual-level α = 0.731. Couple-level α = 0.723.)				
57. Who admitted their …weaknesses to the other?^a, c^	0.50	0.693	0.54	0.664
60. Who talked about their struggles related to friends?^a, b^	0.53	0.671	0.44	0.681
61. Who expressed their feelings more?	0.59	0.634	0.58	0.639
62. Who emotionally needed the other person more?	0.51	0.677	0.54	0.656
Rational (Individual-level α = 0.797. Couple-level α = 0.773.)				
88. Who was considered more “rational” and less emotional?	0.67	–	0.63	–
89. Who used “rationality” to justify their viewpoint?	0.67	–	0.63	–
Spending (Individual-level α = 0.766. Couple-level α = 0.848.)				
74. Who distributed or decided how the money is allocated?^a^	0.63	–	0.75	–
75. Who had the final say about spending money?^a^	0.63	–	0.75	–
Financial needs (Individual-level α = 0.723. Couple-level α = 0.815.)				
59. Who talked about their financial concerns?	0.57	–	0.71	–
67. Who asserted their needs about money more?	0.57	–	0.71	–
Time (Individual-level α = 0.765. Couple-level α = 0.817.)				
94. Who had more time to pursue their interests?^d^	0.63	–	0.70	–
95. Who got to use their time the way he/she wanted to?	0.63	–	0.70	–
Accommodation (Individual-level α = 0.707. Couple-level α = 0.733.)				
81. Who altered their habits and ways of doing things...?^d^	0.55	–	0.58	–
80. Who was more likely to give in to the other’s wishes...?^d^	0.55	–	0.58	–
Avoidance (Individual-level α = 0.681. Couple-level α = 0.802.)				
86. Who was more likely to shut down and not listen?^c, d^	0.34	0.769	0.62	0.761
64. Who kept silent more in disagreement?^d^	0.61	0.422	0.66	0.727
63. Who withheld emotions or avoided conflict more?^d^	0.55	0.513	0.69	0.696
Status (Individual-level α = 0.627. Couple-level α = 0.680.)				
44. Whose occupation is considered higher in status?^d^	0.46	–	0.52	–
45. Who has higher education?^d^	0.46	–	0.52	–
Social (Individual-level α = 0.748. Couple-level α = 0.762.)				
76. Who generally decided whose friends to go out with?^a^	0.63	0.604	0.56	0.723
78. Who generally decided when to see family or relatives?^a, c^	0.49	0.772	0.61	0.668
82. Who influenced the other about which friends to spend time with?^a^	0.62	0.818	0.63	0.646
Children (Individual-level α = 0.837. Couple-level α = 0.792.)				
53. If you have children, who spent more time with them?	0.54	–	0.66	–
54. …who would stay home if the child was sick?^d^	0.54	–	0.66	–

^a^These questions have a high level of *agreement*

^b^Question tends to not load well with the others, but is considered highly predictive and reliable

^c^These questions are more *consistent* and reliable on the *couple* level than the individual level

^d^These questions tend to have a high level of *disagreement*

### Agreement Between Male and Female Partners

While part of the goal was to understand what factors are in relationship balance, another goal was to see which of the questions couples would agree on. Couples tended to agree on most subscales. After reverse-scoring the female partners’ answers to match the direction of the male partners’ answers, they had a positive correlation between their full scale score (r = .49, p < .01) and many subscales. Most of the questions that were highest in agreement were actually dropped from the RBA due to either poor factor loadings or non-normality since many participants would agree by saying the balance was equal. Only four factors had retained items with a high level of agreement: Relational, Emotional Expression, Spending, and Social Choice. (In the factor analysis based on the areas of agreement, money and sex seemed to load consistently together.) Under the Relational subscale, participants typically agreed on who was likely to listen to the other or to proactively maintain a connection or give time to the relationship. With the exception of one item on the relational subscale, about who asks questions, the factors that couples disagreed the most on were: Child Care, Status, Avoidance, Accommodation, Time Discretion, and Spending. The most disagreement was about who had higher status and who was likely to shut down or keep silent during disagreements.

## Discussion

Unless gendered power is clearly and proactively addressed, many couples will remain stuck in entrenched positions. However, power dynamics among intimate partners can be illusive and hard to discern. The Relationship Balance Assessment offers a clinical scale that can help detect the underlying balance of power in relationships. While there have been studies that identify what power and equality look like, this study extended previous qualitative literature to determine whether an inductive procedure could be used to quantify or measure dyadic power, and to distinguish common factors of dyadic power. It is an important step forward in being able to assess relational power processes more broadly.

Past studies tended to measure power in relationships as a one-dimensional concept that resides within the individual. An exploratory factor analysis of the RBA identified 12 common factors that were consistent between men and women and across the individual and couple level. These factors had to do with the relative balance of power in terms of time discretion, relational maintenance, emotional expression and avoidance, accommodation, saving and spending, sexual dominance, economic roles of child care and occupational status, and social decisions. While most of the factors in this study correlated with each other, this study also shows that not all of the subscales correlated highly with each other. This means that the concept of power likely has a multidimensional nature.

The RBA provides therapists and researchers who work with distressed couples a clearer understanding of the array of factors that contribute to processes of power and equality among intimate partners. It includes, but extends beyond, the division of labor and who makes decisions, to incorporate micro-communication and interactional processes that structure daily life and determine whose needs and interests are addressed. To be most effective, the instrument is best used when the responses of one partner are compared to the other and discrepancies identified.

### Factors in Relationship Power

Results indicate that there is a structure of common factors or latent variables underlying the assessment of relationship balance or power. This structure will need to be verified with a follow-up confirmatory factor analysis. There was a surprising degree of similarity between models with only a few minor differences. All of the EFA models presented explain between 51 and 72.3% of the cumulative variance. A review of the literature found that the popular belief is that 75–90% of the variance should be accounted for, though some say as little as 50% is acceptable (Beavers et al. 2013). However, the method may play a role as component analysis tends to explain more variance than methods that only include common variance in the analysis (Beavers et al. 2013), as used in this case. This finding that there are factors in power was expected and is consistent with the literature on equality and power in couples (Jonathan and Knudson-Martin 2012; Knudson-Martin 2013; Marks et al. (2001); Steil 1997).

### TREASURES Acronym

The 12 factors in the Relationship Balance Assessment are summarized in an acronym that clinicians could remember when working with couples (see Table 5). The acronym “TREASURES” was chosen because it is long enough to include 12 subscales and the word is consistent with the researcher’s philosophy and a priori assumption in therapy that humans have intrinsic worth and that equality involves treating others as equally valuable.

**Table 5:** TREASURES acronym

T	Time discretion
R	Relational power
E	Emotional power (expression and avoidance)
A	Accommodation
S	Spending and Saving
U	Union or sexual dominance
R	Rational
E	Economic role power (status vs. child care)
S	Social choices

### Limitations and Clinical Recommendations

There are important limitations that should guide the scope of applying this study’s results. We wish to note that although the initial results show a great deal of promise for the measure, applications and use should be limited until a future confirmatory factor analysis process is completed that verifies the factor structure found in this exploratory study. As this assessment is still in an early stage of development, further tests need to be conducted to replicate these findings in order to ensure that these factors are solid, to establish or confirm their benchmarks, and to confirm its convergent validity. There may be other items or factors that could arise with additional testing. First, while the results appeared reliable, it is based on a relatively small convenience sample of English-speaking couples and may not be generalizable to the larger population. While diverse in race and socioeconomics, participants were also predominantly Christian, and many were Seventh-day Adventist. Overall, couples in this study did not significantly vary in levels of education and household income. Therefore, the factors identified should be confirmed with other populations too, such as different religious groups, lower socio-economic classes, and with same-sex couples.

While the assessment was tested with heterosexual couples, same-sex couples theoretically could use the assessment too, though interpretations would not be gender-based. They would compare their scores to an “equal” score (159–171), and anything significantly different might indicate a distressing inequality. However, it is unknown whether same-sex couples may have different factors that make up relationship balance.

Until the assessment is confirmed with other populations, the use of the measure will remain limited in application. It is the hope that by sharing this quantitative assessment with others now, while specifying its limitations, it would help more professionals to be involved in testing and validating the measure on different populations.

As the RBA was designed to be a clinical measure, further research may need to be conducted on the applicability and transferability of the scale to a clinical environment. Since previous research also suggests that couples are likely to perceive more equity than analysis of their micro-dynamics actually demonstrate (e.g., Knudson-Martin and Mahoney 2009; Sullivan 2006), we cannot know for certain how well this tool actually captures inequities of which the participants themselves may not be aware. The value of this study is that it offers a way to help couples to see common areas in which they may have a power imbalance. Of course, the Relationship Balance Assessment is not the only means of assessing the balance of power. Therapists may want to use other methods as well, such as a qualitative interview like the relational assessment proposed by Knudson-Martin and Mahoney (2009).

There are some other limitations to keep in mind so that this assessment can be followed up with other questions. The RBA does not directly address how partners conceptualize gender roles as the focus is more on behavioral criteria for establishing the balance of power. This was kept separate intentionally to establish a correlational link in later phases of the study. Therefore, therapists and researchers should supplement this assessment with attention to gender roles, by asking questions about what couples believe the role of a man or woman ought to be.

While the role of religion will be investigated in later phases of this study, the RBA did not include questions about the role of religion or spirituality. Another study by Gardner et al. (2008) found that some couples triangulate God as a form of harmful power in their relationship. In addition, women who may feel less powerful than their partners may pray more than their male partners (Gardner et al. 2008). Therefore, additional questions could be asked, such as, “Who prays more for the other person?” Religion may play a role because of how religion often reinforces gender hierarchies (Carneiro 2013). Thus, therapists may want to help clients to explore their own understanding of God (Carneiro 2013).

This study highlights the importance of therapists assessing the attunement within couples. As noted above, averaging the responses from partners can mask important differences in perceptions that predict conflict in the relationship. It is more important to understand how couples agree or disagree with each other. Therefore, therapists should compare answers to see how closely attuned partners are. Another limitation of a balance scale is that it is difficult to ascertain the level of engagement or personal power for the individual apart from the other person. When partners say they are “equal,” it does not indicate whether partners are equally engaged or equally withdrawn, or whether they are equally dominant or equally submissive. That is why it is important to qualify findings as an either-or statement.

It is also important for clinicians to understand that power is not merely a property of an individual, but it is systemic and dyadic. Sutherland and Jeffrey (2016) recommend that therapists look at how power is systemically maintained by societal messages or “discourses” as well as with how partners interact with each other. They invite therapists to consider how social messages get implemented in interactions and to look at how both dominance and subordination reinforce each other (Sutherland and Jeffrey 2016). Socio-cultural attunement is created when therapists explore with their clients how culture impacts their behavior (Pandit et al. 2014). This involves therapists listening for social discourses and for opportunities to link the emotions of their clients to the larger social discourses, which then creates resonance between the therapist and the couple (Pandit et al. 2014).

## Closing

This study sought to see how a diverse group of couples perceive the balance of power in their relationships. This study provides empirical, quantitative evidence for many of the factors described in the qualitative literature about power in relationships, and specifically helps in creating a clinical assessment that can be used with couples. Therapists can know what questions to skip or to focus on. Exploring the factors of power helps to see more clearly the power dynamics underlying relationships, and this can be used in working with couples to provide a roadmap to happiness.

## Appendix: Relationship Balance Assessment

*Directions* Think about your current relationship over the past year. Decide who the questions below apply to more, on a scale of 1–9, with 5 being equal or neutral.

*Note* The author grants permission for the use of the Relationship Balance Assessment in non-commercial research and free clinical assessments of couples, as long as the assessment is provided without charge and this note is duplicated along with a citation of the publication below it. All other rights are reserved.

## Scoring Instructions for the Relationship Balance Assessment

Each subscale is marked out on the RBA assessment and an average can be calculated for each subscale. One may plot both partners’ answers on a balance scale and compare it with the normal or average scores of other couples in the table below. To create a total score, one needs to reverse-score a few of the subscales (Relational, Emotional Expression, and Accommodation) and add them together. A total score for him above 165 (the average for men in this study was 159) means he believes she has more power than him. A total score for her of less than 159 means that she believes he has more power. For her, a normal range is 159–182, with an average total of 160. A score of 140–155 for her is typical for clinically distressed couples. (While this study tested heterosexual couples, same-sex couples could hypothetically use 159–171 as equal. For them, anything below 156 or above 174 might indicate distressing inequality.)

**Table:**

	RBA	Men’s view		Women’s view		Average
	Subscales	Mean	±SD	Mean	±SD	Difference
T	Time	4.95	1.37	4.57	1.70	.40
R	Relational	5.21	.85	5.44	1.17	−.23
E	Emotional exp.	5.73	1.01	5.78	1.23	−.05
	Avoidance	4.65	1.56	4.97	1.72	−.37
A	Accommodation	4.84	1.40	4.89	1.56	−.05
S	Spending	4.51	1.55	4.53	1.83	−.02
	Saving/financial	4.74	1.53	4.88	1.85	−.14
U	Sexual	4.26	1.56	4.34	1.76	−.08
R	Rational	3.64	1.46	3.70	1.57	−.06
E	Status	4.50	2.08	4.00	2.51	.50
	Child care	6.08	1.71	7.02	1.66	−.94
S	Social	5.57	.99	5.38	1.33	.19

A difference score is calculated by taking his (partner A’s) total score and subtracting her (partner B’s) total scores from his. This will get at their level of consensus or attunement about power. Contact the researcher for more information. A difference that is negative (<0) generally reflect happy and attuned heterosexual couples because the man is aware of his power and any imbalances and is attuned to his partner. A difference of 5–15 means that the couple tends to overestimate each other’s power and underestimate their own. Clinically distressed couples may have a high difference score of 16–40, meaning they are not attuned to each other.

Differences can be calculated based on both partners’ total overall score or per subscale. See the table above for subscale averages for the men and women in this study. Then you can compare your couple with these other couples’ differences. A score greater than the absolute value of these couples’ differences may be cause for concern. A score less than their differences is probably within a normal range. Otherwise, negative differences (<0) in the Relational, Emotional, Accommodation and Child Care subscales may be danger areas because partners overestimate their own contributions and minimize the others’ contributions to the relationship. Meanwhile, positive difference scores (>0) in the Time, Avoidance, Spending, Saving/Financial, Sexual, Rational, Status and Social subscales may be danger areas because they minimize their own power and perceive the other as having more power relative to the other’s perception. So there would be more conflict about perceptions of power in these areas.
